# Recent advances in the mechanism, transduction, and function of oxygen sensing in plants

**DOI:** 10.1093/jxb/eraf515

**Published:** 2025-11-22

**Authors:** Isabel Manrique Gil, Susana Ubeda Tomas, Hoang Thong Le, Michael J Holdsworth

**Affiliations:** School of Biosciences, University of Nottingham, Loughborough, LE12 5RD, UK; School of Biosciences, University of Nottingham, Loughborough, LE12 5RD, UK; School of Biosciences, University of Nottingham, Loughborough, LE12 5RD, UK; School of Biosciences, University of Nottingham, Loughborough, LE12 5RD, UK; Fukui Prefectural University, Japan

**Keywords:** ERFVII, hypoxia, MBR1, N-degron pathway, oxygen, Plant Cysteine Oxidase, VRN2

## Abstract

Responding to reduced ambient oxygen is a key component of plant adaptation in natural and farmed environments, and is regulated by oxygen-dependent proteolysis of transducer proteins through the PLANT CYSTEINE OXIDASE (PCO) N-degron pathway. Following the discovery of the core biochemistry of oxygen sensing, associated components and downstream pathways have been uncovered that link diverse stages and mechanisms of growth to the requirement for oxygen sensing. Recent developments in understanding the sensing and transduction of the low oxygen signal have highlighted the importance of hypoxia-regulated processes in root development and response to the environment, and have revealed a key role for cyclic hypoxia in leaf development. Although the pathway is highly conserved in eukaryotes, and important transducer proteins evolved early in land plants, multiple components are under active natural selection related to ambient oxygen levels associated with altitude or humidity. Here, we provide an update focused on recent advances in methodology and mechanistic understanding that highlight the importance of oxygen sensing in plant biology.

## Teaser

Oxygen is sensed through a simple biochemical mechanism. Recent advances have provided novel tools and shown the importance of oxygen sensing for diverse developmental processes, and for the evolution of responses to environmental change.

## Introduction

Oxygen is a central molecule of metabolism, produced in plants, algae, and cyanobacteria by photosynthesis, but also required for aerobic respiration and a multitude of biochemical reactions in plants and animals (Holdsworth and Gibbs, 2020; Holdsworth *et al*., 2025). Reduced oxygen availability (hypoxia) is the central problem of flooding and waterlogging—agriculturally important abiotic stresses that are increasing due to climate change (Bailey-Serres *et al*., 2012), and an essential component of animal physiology, pathology, and environmental adaptation (Bigham and Lee, 2014).

Oxygen sensing in animals occurs through the Hypoxia Inducible Factor (HIF) system, and in plants and animals through the orthologous N-terminal dioxygenase PLANT CYSTEINE OXIDASE (PCO)/cysteamine (2-aminoethanethiol) dioxygenase (ADO) oxygen sensors (Weits *et al*., 2014; Masson *et al*., 2019). These sensors are part of the N-degron pathways that relate the stability of protein fragments produced after protease cleavage (proteoforms) to the nature of the amino terminal residue (Varshavsky, 2024). Destabilizing amino-terminal residues are targets for enzymes of the N-degron pathways that result in recognition by E3 ligases and degradation through the proteasome. In plants, PCOs oxidize the amino-terminal destabilizing cysteine (Cys) residue of proteoform substrates in the presence of molecular oxygen (Weits *et al*., 2014), that allows amino-terminal arginylation by arginyl transferases (ATEs) (White *et al*., 2017), and subsequence ubiquitylation by the PROTEOLYSIS (PRT)6 E3 ligase (Fig. 1) (Zubrycka *et al*., 2023). Under reduced ambient oxygen levels, PCO enzyme activity is inhibited, and substrates become stable (White *et al*., 2018). The most established examples of PCO substrates in plants are the Group VII ETHYLENE RESPONSE FACTOR (ERFVII) transcription factors, that become stable in hypoxia and enhance hypoxia-related gene expression (Gibbs *et al*., 2011; Licausi *et al*., 2011). Two other substrates, VERNALIZATION (VRN)2 (Gibbs *et al*., 2018) and LITTLE ZIPPER (ZPR)2 (Weits *et al*., 2019) have also been identified. All these substrates are part of a group of proteins that initiate Met-Cys, a combination that is restricted in proteomes of diverse organisms (e.g. in *Arabidopsis thaliana* there are only ∼200 Met-Cys proteins encoded in the genome) (Gibbs *et al*., 2014a). Amino-terminal Cys is exposed in these proteins following the co-translational action of METHIONINE AMINOPEPTIDASE (MetAP) that removes the initiating Met (Ross *et al*., 2005). Interestingly, in addition to oxygen sensing, the PCO N-degron pathway also senses another gas, nitric oxide (NO), important for response to stresses including drought and salinity (Vicente *et al*., 2017), although it is not known where within the pathway this occurs (Gibbs *et al*., 2014b) (Fig. 1).

**Fig. 1.**
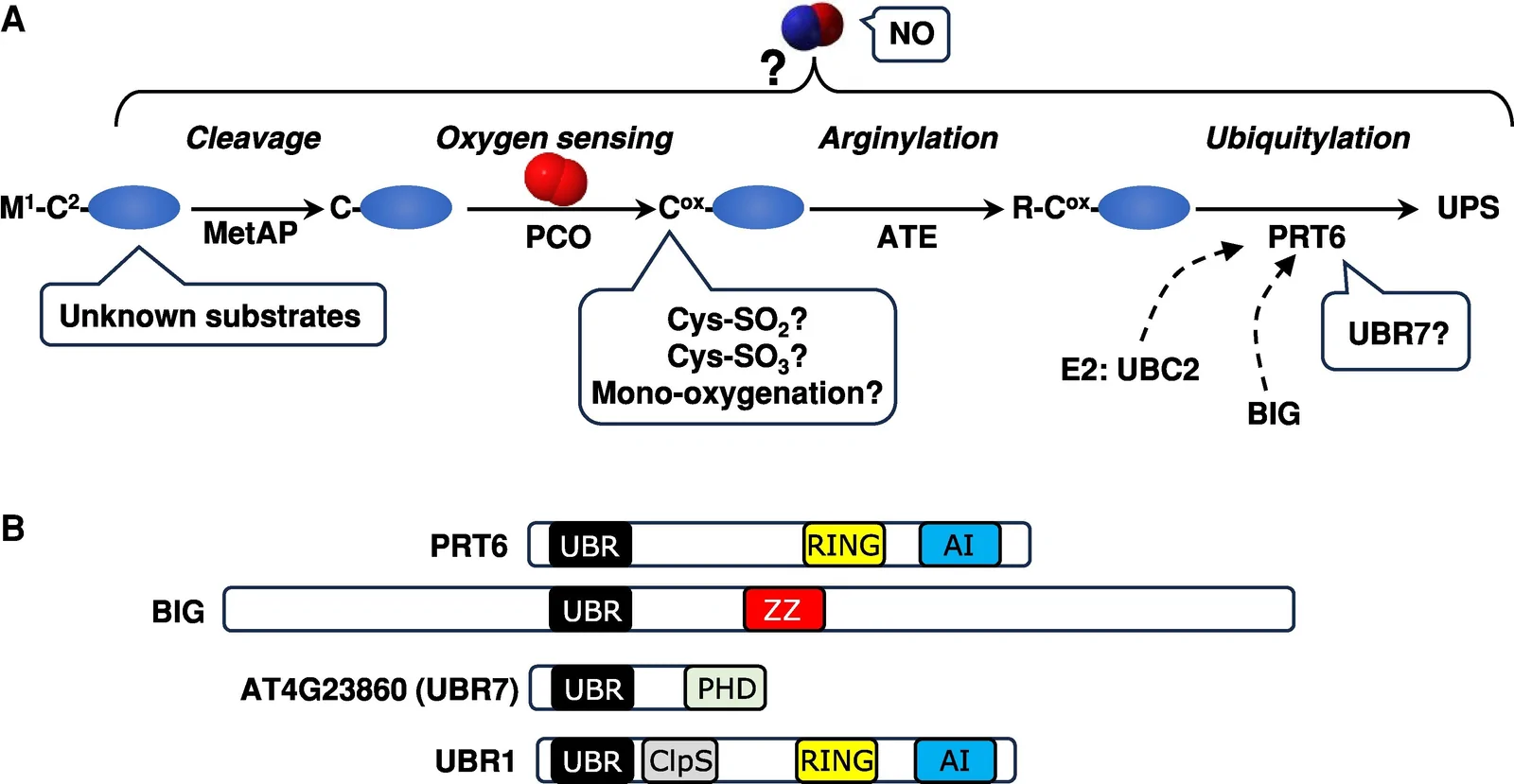
Oxygen sensing through the PCO N-degron pathway. (A) Representation of the PCO N-degron pathway. Open questions in bubbles include the position of NO sensing within the pathway, the level of Cys oxygenation, the possible role of UBR7 in substrate recognition, and the presence of as yet undiscovered substrates. ATE, ARGINYL TRANSFERASE; MetAP, METHIONINE AMINO PEPTIDASE; PCO, PLANT CYSTEINE OXIDASE; PRT6, PROTEOLYSIS 6, UBC2/7, UBIQUITIN CONJUGATING ENZYME 2/7; UPS, ubiquitin proteasome system. Single amino acid codes are used. Ox indicates oxidized Cys. (B) UBR-box-containing protein from *A. thaliana* showing the positions of important functional domains; AI, auto-inhibitory; PHD, Plant HomeoDomain; ZZ, ability to bind two zinc ions.

Since the discovery of the plant oxygen sensing mechanism 15 years ago (Gibbs *et al*., 2011; Licausi *et al*., 2011), great progress has been made both in the development of tools and in understanding how such a simple biochemical mechanism is used by plants to respond to internal ‘chronic’ hypoxia or external ‘acute’ ambient hypoxia. This has led to the conclusion that although environmentally-imposed hypoxia can have very negative effects on plant survival, response to internally generated hypoxia can also form a necessary normal part of plant growth and development. Open questions in the field include: defining the diversity of substrates of the pathway; how these substrates transduce the low oxygen signal to intracellular and physiological responses; how sensing is integrated into the normal life cycle; and how oxygen sensing has influenced the evolution of plants in both natural and agricultural environments. In this review, we focus on exciting recent work in this field, underpinned by the development of novel tools that have highlighted the importance of oxygen sensing in plant growth and response to the environment, and have begun to address these questions (Box 1).

Box 1. **Key developments in understanding the transduction of oxygen sensing**

- **Components and function of the core PCO N-degron pathway:**

Zhang *et al*. (2024) showed that the UBR-box containing protein BIG (also known as DARK OVEREXPRESSION OF CAB1/TRANSPORT INHIBITOR RESPONSE3) (Fig. 1B) acts as an accessory component to the N-degron pathway E3 ligase PRT6, enhancing its capacity to destabilize substrates. *PRT6* and *BIG* were shown to together enhance gene expression and deposition of suberin in the root, that is inhibited through ERFVIIs. A novel role in cyclic hypoxia was discovered for ERFVII-regulated hypoxia gene expression (Triozzi *et al*., 2024). Fluctuations in diurnal hypoxia-regulated gene expression were shown to match carbon status with leaf growth, demonstrating a role for hypoxia in the regulation of normal development of leaves.

- **Evolution of the response to low oxygen:**

An evolutionary analysis revealed that ERFVII appeared as PCO N-degron pathway substrates in vascular plants with true roots, coinciding with the acquisition of many of the ‘core hypoxia-response genes’ (Dalle Carbonare *et al*., 2025) (Fig. 2).

The co-regulator MED25 and the E3 ubiquitin ligase MBR1, which controls MED25 stability, have both been identified as key components of hypoxia signaling. This mechanism is conserved across angiosperms, and has contributed to within-species adaptation to humid environments. (Castellana *et al*., 2024; Schippers *et al*., 2024).

Oxygen sensing is key for altitude adaptation: high-altitude populations have increased oxygen sensitivity and reduced relative ERFVII activity, enabling growth under constant low partial pressure of oxygen (Abbas *et al*., 2022).

- **Technological advances in investigating low oxygen biology:**

Two hypoxia-activated fluorescent probes have been described for mapping low oxygen regions in *A. thaliana* tissues, effectively detecting physiological hypoxic niches and transient hypoxia induced by environmental stress (Perri *et al*., 2025).

**Fig. 2.**
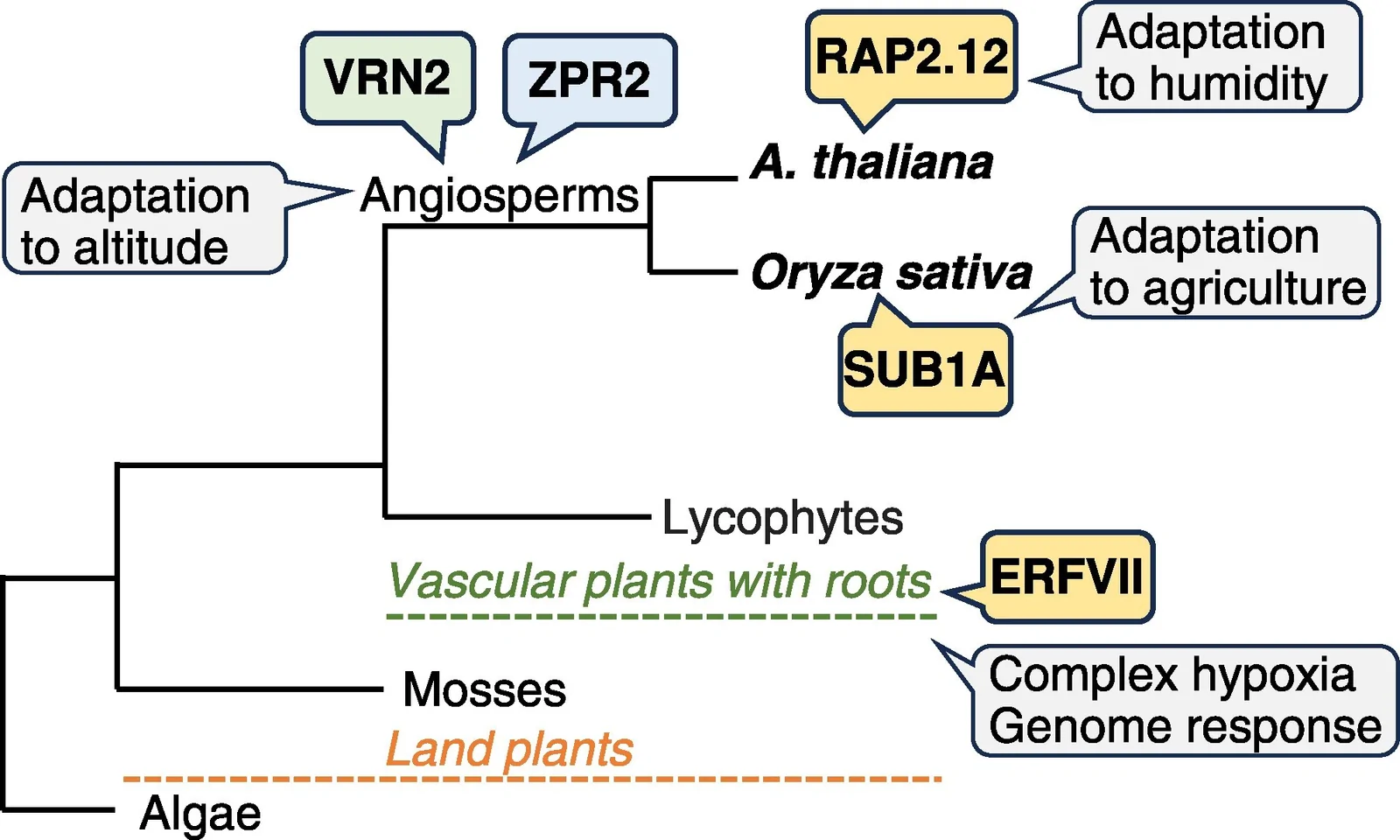
Evolution of different substrates and functions in land plants: Simplified schematic of the evolutionary trajectory of land plant groups. The positions of first appearance of Met-Cys initiating protein substrates of PCO are indicated as colored bubbles. Also shown are the positions of functional adaptations associated with oxygen sensing through ERFVII substrates.

## Sensing and response to low oxygen stress

Oxygen (and NO) sensing through PCOs requires the downstream activity of enzymes of the N-degron pathway, resulting in destruction of transducer substrates via the ubiquitin proteasome system (UPS) (Fig. 1A). Although the essential enzymatic components of the pathway have been identified, several important aspects of the function and outputs of this pathway remain unexplored or not completely understood. For example, although PCO activity results in the production of Cys-sulfinic acid (i.e. both atoms of molecular oxygen transferred to N-terminal Cys), it has been shown that in both mammalian and plant extracts, oxidized Cys exists as Cys-sulfonic acid (i.e. three oxygen atoms attached to N-terminal Cys) (Kwon *et al*., 2002; Zubrycka *et al*., 2023). This suggests that additional enzymes that may comprise mono-oxygenation activity, may also be part of the core sensing pathway (Fig. 1).

The role of the E3 ligase PRT6 [probably in concert with the E2 conjugating enzyme UBIQUITIN-CONJUGATING (UBC)2; Rudi *et al*., 2025] as the only component linking PCO activity to ubiquitin-mediated substrate destruction, was recently challenged (Fig. 1A). It was shown that stability of the ERFVII, RELATED TO AP2 (RAP)2.3, was greater in *A. thaliana* seedlings treated with the proteasome inhibitor bortezomib than in the *prt6* mutant (Zubrycka *et al*., 2023). This suggested that other activities may influence N-degron pathway-regulated stability. Three proteins in the *A. thaliana* genome contain a UBR domain similar to that found in the yeast and human N-degron pathway E3 ligase UBR1, which are important for the recognition of destabilizing residues. These include PRT6, BIG (Holdsworth *et al*., 2020), and the recently described UBR7 (AT4G23860) (Kim *et al*., 2025) (Fig. 1B). In an important addition to the field, it was shown that BIG acts as an accessory factor for PRT6 action (Zhang *et al*., 2024). It was observed that in *big* mutant backgrounds, stability of the PCO substrates ERFVII HYPOXIA-RESPONSIVE ERF2 (HRE2) and VERNALIZATION (VRN)2 was not altered, but that in a background combining *big* and *prt6* mutations, accumulation of substrates was greater. This increase in stabilization was mirrored by a corresponding increase in ERFVII-regulated hypoxia gene expression and tolerance to hypoxia. These results suggest that BIG acts as an accessory component within the PCO N-degron pathway, perhaps by enhancing the activity of PRT6, though it is not required for PRT6 action. Thus far, no role for UBR7 in regulating the stability of PCO substrates has been assessed, and it is interesting to speculate that this protein could also act with PRT6 and BIG in increasing destabilization of PCO substrates.

A key function of ERFVIIs is the enhancement of tolerance to acute hypoxia (reduced ambient oxygen conditions), demonstrated for example, by the strong reduction in root growth observed in plants without ERFVII genetic activity in response to waterlogging (Zubrycka *et al*., 2023). In addition, understanding the influence of chronic, internally generated hypoxia is gaining increasing interest. For example, it was shown that hypoxia at the shoot apical meristem enhances the stability of the PCO substrates VRN2 and ZPR2, influencing their function in growth and development (Weits *et al*., 2019; Labandera *et al*., 2021; Osborne *et al*., 2025). As plants produce oxygen through photosynthesis, it would be expected that PCO substrates would be unstable, and therefore non-functional in green tissues. However, a recent study demonstrated that the hypoxia response of young emerging leaves shows cyclic diurnal fluctuations, with hypoxia-responsive genes activated, and ERFVIIs stabilized during the night (Triozzi *et al*., 2024). It was proposed that this is the result of increased energy demand in growing tissues during the night, and that activation of the hypoxia response allows control of leaf growth in response to changing carbon status.

An additional mechanism for generating chronic hypoxia could be through tissue-level impediment of oxygen entry. During secondary root growth, a periderm layer is established, with suberin deposition as an outer protective tissue, and is regenerated following wounding. A recent study showed that the vascular region of mature *A. thaliana* roots is under a slightly reduced oxygen tension that results in slightly enhanced expression of the ERFVII-regulated *PCO1* gene (Iida *et al*., 2025). Following wounding, oxygen tension increases and *PCO* expression decreases. Oxygen sensing was shown to influence periderm formation and suberization in relation to wounding. Regeneration of a suberized layer was inhibited in the *ate1 ate2* mutant that does not contain arginyl transferase activity, although this was not the case in the *prt6* background. Ethylene augmented this effect, and interestingly, ethylene was previously shown to regulate stabilization of ERFVIIs by enhancing depletion of NO (Hartman *et al*., 2019). Intriguingly, it was also shown that *PRT6* and *BIG* interact to enhance suberin-related gene expression and deposition in untreated roots, which are repressed in an ERFVII-dependent manner (Zhang *et al*., 2024), although this was not investigated in wound periderm formation. These studies demonstrate an important emerging role for N-degron pathway regulation of oxygen sensing in root biology.

Downstream of oxygen sensing, the signals are transduced through stabilized substrates. An important open question relates to the identity of these substrates of the PCO N-degron pathway (Fig. 1A). At present, only three have been found in plants, and all are Met-Cys initiating proteins—ERFVIIs, VRN2, and ZPR2 (Fig. 2). All of these have transcription-associated functions, and therefore, transduce the low oxygen signal via genome-wide changes in gene expression. Interestingly, none of the three identified groups of mammalian Met-Cys initiating substrates are nuclear-localized (Hu *et al*., 2005; Lee *et al*., 2005; Masson *et al*., 2019; Shim *et al*., 2023). A major goal of future work will be to determine which of the other Met-Cys encoded proteins within plant genomes, or proteins internally cleaved by endopeptidases to reveal amino-terminal Cys, are transducers of oxygen (and NO) sensing. Importantly, not all proteins that initiate Met-Cys are substrates of PCO, including MADS AFFECTING FLOWERING (MAF5) (Gibbs *et al*., 2011), AP2-LIKE ABA REPRESSOR (ABR)1 (Bäumler *et al*., 2019), and NIN-LIKE PROTEIN (NLP)7 (Castillo *et al*., 2021), for conformational or other reasons [including the rice ERFVII SUBMERGENCE (SUB)1A used in breeding programmes to enhance flood tolerance; Lin *et al*., 2019]. Detecting true PCO substrates is not an easy endeavor, as shown by the small number thus far identified. Novel substrates may contribute to other, as yet undiscovered, roles of oxygen sensing in plant biology. In addition, it should be noted that the existence of other oxygen sensing mechanisms unrelated to PCOs and their substrates, has been discussed previously (Holdsworth, 2017; Wang *et al*., 2017).

## Evolution of the response to low oxygen stress in land plants

Recent publications have expanded our knowledge of how oxygen sensing evolved in land plants, illustrating the diversification of the hypoxia response machinery, which is tightly linked to environmental pressures across plant species and habitats (Fig. 2). The core of this sensing is the ERFVII transcription factors, and an evolutionary analysis showed that these first appeared as hypoxia-regulated transducers, substrates of the PCO N-degron pathway, in vascular plants with true roots (in present day represented by tracheophytes) (Dalle Carbonare *et al*., 2025). The emergence of ERFVIIs in vascular plants correlates with the acquisition of many genes from the ‘core-hypoxia response’ identified in *A. thaliana* (Mustroph *et al*., 2009), and overlaps with a strong N-terminal sequence conservation, specifically the Cys2 residue and gradual fixation of residues immediately C-terminal to this. Interestingly, other Cys2-ERFs (from groups outside VII) are present as PCO substrates multiple times in different plant lineages (Dalle Carbonare *et al*., 2025).

Ecological pressures, such as adaptation to rainy environments or high altitude, have shaped the diversification of hypoxia signaling in more recently derived plant lineages. New regulatory layers of the hypoxia response have been discovered with the introduction of two new players: the co-regulator MEDIATOR SUBUNIT 25 (MED25) (Schippers *et al*., 2024), and the E3 ubiquitin ligase, MED25 BINDING RING-H2 PROTEIN (MBR1) (Castellana *et al*., 2024). The *A. thaliana* MED25 is a nuclear co-regulator of ERFVIIs. Under low-oxygen stress, it interacts with the ERFVII members RAP2.2 and RAP2.12, facilitating RNA polymerase II recruitment to gene promoters containing hypoxia-responsive promoter elements (HRPEs), allowing the transcription of hypoxia-responsive genes like *PYRUVATE DECARBOXYLASE* (*PDC*)*1*, *LATERAL ORGAN BINDING DOMAIN* (*LBD*)*41* and *STERAROYL-ACYL CARRIER PROTEIN (SAD)6* (Schippers *et al*., 2024). Notably, the MED25–ERFVII interaction is conserved in both *A. thaliana* and rice, suggesting that this mechanism arose early in angiosperms. This co-regulatory module is integrated with non N-degron-mediated post-translational modification of the hypoxia system. MBR1 is an E3 ubiquitin ligase that controls MED25 stability (Castellana *et al*., 2024). Using environmental genome-wide association studies (eGWAS) across 934 *A. thaliana* ecotypes, it was found that natural variants of *MBR1* are enriched in populations from rainy environments. Within these environments, accessions carrying non-functional *MBR1* alleles exhibit increased MED25 stability, which enhances the transcriptional response to low oxygen (Castellana *et al*., 2024). Thus, the recruitment of a general transcriptional co-regulator and the proteasomal degradation of this co-regulator are coupled to oxygen sensing, and have contributed to plant adaptation to rainy environments.

Changes in *cis*-regulatory elements are another mechanism for adaptation to low oxygen. Two allelic variants in promoter motifs of *RAP2.12*, namely the W-box and WT-box, determine tolerance to flooding or drought in *A. thaliana* (Lou *et al*., 2022). Whereas the W-box is the ancestral variant, conserved across *Brassicaceae* species, the WT-box haplotype evolved after *A. thaliana* diverged, facilitating the colonization of humid regions. The transcription factor WRKY70 binds specifically to the WT-box, enhancing *RAP2.12* expression in response to submergence or flooding. A similar regulatory mechanism involves *WRKY33*, where WRKY70 binds to two identical *cis*-elements located in the promoter and intron of *WRKY33* in *A. thaliana* accessions from humid areas (Liu *et al*., 2024). As a result, *WRKY33* expression is boosted compared with accessions from dry regions, which only possess the *cis*-element in the intron. The higher expression of *WRKY33* results in a more robust activation of the hypoxia-response genes through direct DNA binding, alongside WRKY12, to the W-box *cis*-element in *RAP2.2* promoter (Liu *et al*., 2021; Tang *et al*., 2021).

Finally, it was recently shown that low oxygen sensing has a greater adaptative value beyond flooding stress. Direct oxygen sensing was shown to be key for angiosperm adaptation to absolute altitude, based on ambient oxygen partial pressure (*pO_2_*) (Abbas *et al*., 2022). Populations adapted to the low ambient O_2_ present at high altitude have an increased O_2_ sensitivity. By thereby exhibiting reduced ERFVII stability, these populations can survive in environments with chronic low oxygen availability and produce the same physiological and metabolic response as populations from low altitudes, preventing a constitutive response to hypoxia.

Taken together, these recent studies show that the ancient ERFVII oxygen-sensing module has been optimized during evolution with novel regulatory layers, involving the addition of transcriptional co-regulators, repressors, ubiquitin-mediated protein turnover, and diversification of *cis*-regulatory elements (Fig. 2).

## Technological advances in studying low oxygen stress

Advances in methodologies and tools to directly measure oxygen levels and changed cellular states that are consequences of oxygen sensing, greatly aid understanding of how oxygen is sensed and integrated into cell and developmental biology. A new staining technique for visualizing N-terminal reduced cysteines in animal cells was recently published (Choi *et al*., 2025). This method is based on the *De Novo* Formation of a Citrate-based fluorophore (termed DNFC), thiazolopyridine (TPA), which is specifically generated via condensation between citrate and L-cysteine. Tissues treated with non-fluorogenic citrate and a coupling reagent that facilitates the reaction under physiological conditions, yields a robust cyan fluorescence wherever there is an N-terminal Cys residue. This new approach is highly selective; TPA formation requires both an α-amino and β-thiol group, making the reaction specific to reduced N-terminal Cys residues. Moreover, the fluorescence intensity shows a linear dependence on Cys concentration. The DNFC staining is effective in various mammalian tissues, and could be potentially applied to plant tissues. The cyan fluorescence should easily be distinguished from chlorophyll autofluorescence, but other technical limitations may arise in its use, for example cell wall barrier, a restricted use to only softer tissues such as aerial or younger tissues, plant-specific metabolic activity, redox state variations or protein abundance. If optimized for plants, this staining technique could allow spatial mapping of hypoxic regions, and the identification of new oxygen-sensitive proteins or ‘hypoxia biomarkers’ that expose N-terminal Cys as a result of the N-degron pathway inhibition and endopeptidase activity during hypoxia.

While DNFC staining represents a promising technique at the protein level, complementary approaches are emerging to map low-oxygen environments *in planta*. A recent study successfully translated two hypoxia-activated fluorescent probes previously published in bacterial and mammalian cells (Collins *et al*., 2018; Wallabregue *et al*., 2023) to *A. thaliana* (Perri *et al*., 2025). The probes 4NB-resorufin and MeIQ-resorufin were effective in detecting both physiological chronic and acute stress hypoxia in *A. thaliana*. These probes are irreversibly reduced by plant reductases when oxygen levels are limited, releasing the fluorescent resorufin, thereby allowing precise visualization of low-oxygen regions. The MeIQ-resorufin shows greater sensitivity to mild hypoxia and faster response kinetics than 4NB-resorufin. Both DNFC staining and hypoxia-activated probes are examples of non-invasive techniques that do not require previous genetic manipulation.

Finally, it is worth mentioning the utility of genetically encoded fluorescent protein sensors to study hypoxia-induced metabolic changes. They have been successfully applied in *A. thaliana*, where multiple cytosolic parameters (Mg-ATP depletion, alteration in Ca^2+^ dynamics, pH, NAD redox and glutathione redox) were monitored in parallel in leaves during progressive O_2_ depletion (Wagner *et al*., 2019). This approach allowed the visualization of cytosolic changes during hypoxia, where inhibition of the mitochondrial electron transport was determined to be the main factor of cellular changes. Changes were also linked to transcriptional responses, providing an integrated framework that connects immediate metabolic state to transcriptional responses during hypoxia.

## Conclusion

Oxygen sensing is a component of both plant responses to the environment, and normal development. Recent publications have shown the diversity of roles played by oxygen sensing in plant biology, by defining novel components and processes that are directly influenced by ambient and internal oxygen levels. Emerging technologies will aid the understanding and investigation of how oxygen levels vary through development, whilst discovery of novel substrates may provide clues to other important functions related to adaptation to hypoxia. This is an emerging field of demonstrably increasing importance within plant biology. Many open questions remain, even for the core sensing mechanism, that will be interesting and important targets for investigation in the coming years.

## Data Availability

This work does not include any new data.
